# Wood chemical composition of forest management residues for bioenergy

**DOI:** 10.1007/s00226-026-01760-z

**Published:** 2026-03-07

**Authors:** Samuel Roy Proulx, Évelyne Thiffault, Derlly Julieth Ortiz Niño, Claudie-Maude Canuel, Nelson Thiffault, Véronic Landry

**Affiliations:** 1 Département des sciences du bois et de la forêt, Centre de Recherche sur les Matériaux Renouvelables (CRMR), Université Laval, Québec, Canada; 2 Service canadien des forêts, Ressources naturelles canada, 1055 rue du P.E.P.S, C.P. 10380, Succ. Sainte-Foy, Québec, QC G1V 4C7 Canada

## Abstract

We studied the suitability of forest management residues as feedstock for bioenergy production. We focused on the variability of their chemical and physical properties that are most likely to influence the efficiency of thermochemical conversion and the factors underlying this variability, namely wood species, stage of wood decomposition, and the site characteristics of the source location. We used wood from leftover logs and large branches of birch (*Betula papyrifera* and *Betula alleghaniensis*) and balsam fir (*Abies balsamea*), which are abundant in the boreal and mixedwood forests of Quebec (Eastern Canada) and likely to be found as residues on cutblocks. We used factor analysis of mixed data and linear mixed models to explore the relationships among wood species, decomposition class, chemical concentrations and density, and site characteristics such as bioclimatic conditions and soil cation exchange capacity. Model results showed significant impacts of species interacting with bioclimatic domains, notably on net calorific value, wood base cations (calcium, magnesium and potassium) and carbon concentrations. Balsam fir had the highest net calorific value (ranging from 17.0 to 17.4 MJ kg^−1^) compared with birch (16.3 to 17.0 MJ kg^−1^) in two of the three studied bioclimatic domains. However, balsam fir potassium concentration was doubled compared to birch (593 vs 245 ppm respectively) in one bioclimatic domain, which can be undesirable for thermochemical conversion. The wood decomposition class was effective at predicting significant decreases in wood chemical components (potassium, cellulose and hemicellulose concentrations). Our results suggest that forest management residues from balsam fir and birch could be suitable for biomass procurement, providing insights into their potential for bioenergy production in eastern Canada.

**Supplementary Information:**

The online version contains supplementary material available at 10.1007/s00226-026-01760-z.

## Introduction

Modern bioenergy conversion systems based on forest biomass are considered important renewable energy sources for reducing reliance on fossil fuels and mitigating greenhouse gas (GHG) emissions (Bashmakov et al. 2022; Shukla et al. 2022). Numerous studies have highlighted the opportunities, challenges, and constraints of large-scale forest biomass use as a substitute for fossil fuels (Goyal et al. 2008; Yu et al. 2021). A key advantage of forest biomass is its versatility, as it can be converted into solid, liquid, or gaseous energy carriers (Hoefnagels et al. 2014).

The conversion of forest biomass to bioenergetic products can follow the biochemical or the thermochemical pathways. While biochemical conversion pathways include processes that involve degradation (that can be induced artificially) through the action of enzymes or bacteria, thermochemical conversion pathways use heat as a vector of decomposition of the feedstock (McKendry 2002). The thermochemical conversion of forest biomass, through processes such as combustion, pyrolysis, and gasification, makes it possible to generate products that can substitute fossil fuels such as fuel oil, coal, and natural gas (Shah and Kaur 2024). For their part, biochemical processes such as hydrolysis and fermentation can produce biofuels such as bioethanol (Ayala-Mendivil and Sandoval 2018; Vaillancourt et al. 2019). Thermochemical conversion is particularly relevant in Canada, as it can be implemented on a larger scale nationwide. Indeed, several thermochemical pathways are already technologically mature for forest biomass conversion (Pang 2019). For example, wood biomass combustion can be used as a primary heat source in community networks, and pyrolysis appears as one of the most promising technologies for the production of biofuels, biochar and high‑value chemicals (Vaillancourt et al. 2019).

Despite its well-established forest sector, Canada’s forest-based bioenergy industry remains underdeveloped, accounting for only 8% of its total energy production (Vaillancourt et al. 2019). Forest biomass sources include residues from forest management and sawmill processing (Barrette et al. 2015; Paré et al. 2011, 2016). The latter, comprised of wood chips and shavings, is often already fully utilized as feedstock for internal heat production within mills and/or the production of pulp or particle boards (Ghafghazi et al. 2017). On the other hand, residues from management of Canadian forests are largely underutilized. Their availability has been estimated at 14 ± 2 oven-dried megatonnes of biomass per year (Mansuy et al. 2017). These residues consist of bucking and trimming materials, tree tops, branches, stem sections, and other materials left on harvest cutblocks (Thiffault et al. 2015).

The quantity and species composition of biomass available as forest management residues for bioenergy production depend on regional wood procurement strategies and the existing industrial network focused on conventional wood products i.e., sawtimber, pulp and panels (Canuel et al. 2024, 2025a, b; Durocher et al. 2019). Therefore, bioenergy conversion systems must adapt to the regional variability in biomass supply, notably shaped by forest management practices and market demands (Benjamin et al. 2009; Canuel, et al. 2025a, b). For example, species of the *Betula* genus, including *Betula papyrifera* Marsh. and *Betula alleghaniensis* Britton are hardwood species often found in boreal (for *B. papyrifera*) and temperate (for *B. alleghaniensis*) mixedwood stands of eastern Canadian forests. In Quebec, their wood is often underutilized in areas without pulp and paper mills (Durocher et al. 2019), and it is therefore left on cutblocks. Balsam fir (*Abies balsamea* (L.) Mill.) is a shade-tolerant softwood species prevalent in boreal and mixed forests of Eastern Canada; in Quebec, it is the second most harvested conifer after black spruce (*Picea mariana* (Mill.) B.S.P.) (Lemay et al. 2022). Despite its abundance, balsam fir is vulnerable to root rot (Whitney 1989) and to periodic spruce budworm (*Choristoneura fumiferana* (Clem.)) outbreaks that cause widespread defoliation (Blais 1983), degrading the quality of its fibre and reducing its suitability for sawtimber and pulp production (Lemay et al. 2022). Wood from birch and balsam fir is likely to represent a large share of forest management residues currently found on cutblocks of eastern Canada and therefore available for bioenergy production.

The suitability of wood biomass for energy depends on its chemical composition (Sengupta and Pike 2012; St-Pierre et al. 2013). Understanding wood chemical composition and physical properties is essential for ensuring its efficient thermochemical conversion into energy products (Gendek et al. 2023). The energy conversion efficiency of thermochemical processes is particularly influenced by the relative amounts of lignin, cellulose, and hemicellulose in the wood biomass feedstock (Moya and Tenorio 2013; Wang et al. 1982; White 1987). Since lignin has a higher energy content per unit mass than cellulose, wood species with higher lignin concentrations generally exhibit higher calorific values (Moya and Tenorio 2013; Shmulsky and Jones 2019; Wang et al. 1982; White 1987). For example, lignin concentrations are generally higher in softwood trees species, ranging from 25 to 33%, than in hardwood species, in which they fluctuate from 18 to 34% (Quintana et al. 2024).

Specific chemical components of biomass can negatively affect thermochemical processes (Elbersen et al. 2015, 2017). Elements such as nitrogen (N), potassium (K), magnesium (Mg), and calcium (Ca) can cause issues like reactor sintering, boiler fouling, and the release of undesirable gases. High biomass N concentrations, for instance, can increase NOx emissions, necessitating costly emission control systems, while high K concentrations can contribute to fouling in thermal reactors (Elbersen et al. 2015, 2017).

Wood properties are influenced by multiple factors, including tree species, forest stand characteristics (such as age and tree density), soil fertility and climate (Tharakan et al. 2003; Waliszewska et al. 2019). Even within a single species, the chemical composition of biomass can vary across regions, possibly affecting its suitability as feedstock for bioenergy production. Moreover, ecological processes such as wood decomposition, primarily driven by fungal activity, can significantly alter wood chemical composition and influence its calorific value (Gendek et al. 2023; Piętka et al. 2019; Vidholdová et al. 2022).

In this study, we assessed the suitability and variability of forest management residues as feedstock for bioenergy production. As a case study, we used residues (mainly leftover sections of stem wood and large branches) from balsam fir and birch collected on harvest cutblocks of Quebec. Our specific objective was to characterize the variability of their chemical and physical properties and to identify the factors underlying this variability, namely wood species, degree of wood decomposition, and the site characteristics of the source location of wood, including its bioclimatic conditions and soil properties. We concentrated on wood properties most likely to affect the efficiency of thermochemical conversion to bioenergy.

## Materials and methods

### Study area

**Fig. 1:**
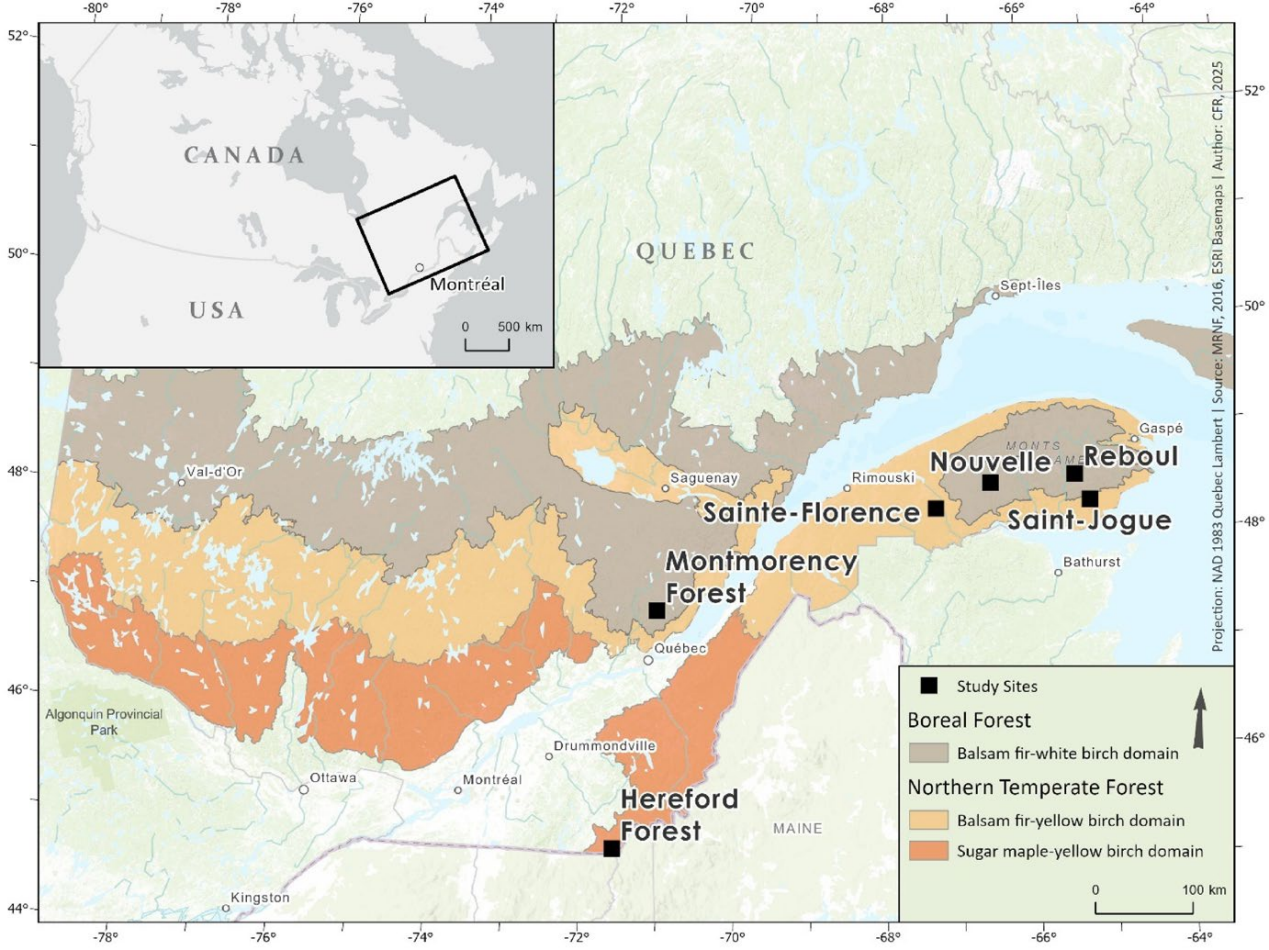
Location of study sites distributed in three bioclimatic domains in Québec, Canada (Saucier et al. 2009), namely the balsam fir (*Abies balsamea*)–white birch (*Betula papyrifera*) (Nouvelle, Reboul, Montmorency Forest), the balsam fir-yellow birch (Sainte-Florence, Saint-Jogue) and the sugar maple (*Acer saccharum*)-yellow birch (*Betula alleghaniensis*) (Hereford Forest)

The study sites were those used in Canuel et al. (2024), located in southern Québec, Canada. All sites were subjected to harvesting and were located in the Gaspésie (Sainte-Florence [lat.48° 25' N, long. 67° 29' W]; Nouvelle [lat. 48° 29' N, long. 66° 31' W]; Saint-Jogue [lat. 48° 32' N, long. 65 20' W]; Reboul [lat. 48° 17' N, long. 65°09' W]), Eastern Townships (Hereford Forest [lat. 45° 06' N, long. 71°59' W]) and Capitale-Nationale (Montmorency Forest [lat. 47° 27' N, long. 71°12' W]) regions (Fig. 1). These sites are located within the balsam fir–white birch, balsam fir–yellow birch and sugar maple (*Acer saccharum* Marsh.)–yellow birch bioclimatic domains, as classified by Saucier et al. (2009) (Fig. 1). Bioclimatic domains are defined as large ecological regions characterized by relatively homogeneous climate and soil conditions, which in turn determine the distribution of natural vegetation (Saucier et al. 2009). In this study, bioclimatic domains were considered to account for the ecological context associated with broad-scale climatic gradients and to detect potential species differences linked to regional conditions.

Mean annual temperature and precipitation, recorded at the nearest weather stations, varied between sites and ranged from 0.5 to 5.8 ºC and 957 to 1583 mm yr^−1^, respectively (Table 1; Environment Canada 2024). The surficial deposits at the studied sites primarily consisted of glacial tills. Soils at Montmorency Forest, Nouvelle, Reboul, Saint-Jogue and Sainte-Florence were classified as ferro-humic or humo-ferric podzols, and soils at Hereford Forest, Saint-Jogue, and Sainte-Florence included melanic, sombric or dystric brunisols (Canuel et al. 2024). To assess and compare site properties, we extracted the cation exchange capacity (CEC) at a soil depth of 15–30 cm from SIIGSOL‑100 m, a 100 m‑resolution predictive soil mapping product for non‑urban areas available on the portal '*Données Québec*' (MRNF 2022). CEC was selected because it is a well‑established proxy for soil fertility, indicating the capacity of soils to retain and supply essential plant nutrients, such as Ca^2^⁺, Mg^2^⁺ and K⁺, which directly influences vegetation growth and overall site productivity (Manrique et al. 1991).

**Table 1:** Study site (Hereford Forest, Montmorency Forest, Reboul, Nouvelle, Sainte-Florence and Saint-Jogue) description with geographical coordinates in Québec, Canada, bioclimatic domains (SM—YB, the sugar maple (*Acer saccharum*)-yellow birch (*Betula alleghaniensis*), BF—YB, balsam fir (*Abies balsamea*)–yellow birch (*Betula alleghaniensis)*, BF—WB, balsam fir (*Abies balsamea*)–white birch (*Betula papyrifera*), mean annual precipitation and temperature, stem density before harvest from Canuel et al. (2024) (Table S10), and cation exchange capacity (CEC) in the 15–30 cm soil layer

Sites (coordinates)	Bioclimatic domains	Mean annual precipitation (mm yr^−1^)	Mean annual temperature (ºC)	Stand density before harvest (stem ha^−1^)	CEC (mol kg^−1^)
Hereford Forest (45°06′N, 71°59′W)	SM—YB	1216	5.8	845	2.75
Montmorency Forest (47°27′N, 71°12′W)	BF—WB	1504	0.6	1890	2.30
Sainte-Florence (48°25′N, 67°29′W)	BF—YB	1003	2.7	1804	4.84
Saint-Jogue (48°32′N, 65°20′W)	BF—YB	957	4.0	1100	5.18
Reboul (48°17′N, 65°09′W)	BF—WB	957	4.0	1369	5.61
Nouvelle (48°29′N, 66°31′W)	BF—WB	1203	1.9	2608	3.79

The main deciduous species at the study sites included sugar maple, red maple (*Acer rubrum* L.*),* yellow birch, black cherry (*Prunus serotina* Ehrh.), American beech (*Fagus grandifolia* Ehrh.), white birch, pin cherry (*Prunus pensylvanica* L. f.), American mountain ash (*Sorbus americana* Marsh.) and trembling aspen (*Populus tremuloides* Michx.). Coniferous species were dominated by balsam fir*,* red spruce (*Picea rubens* Sarg.)*,* white spruce (*Picea glauca* (Moench) Voss) and black spruce*.*

### Sampling method and sample processing

In 2021, we collected wood samples from forest management residues of balsam fir and white and yellow birch (pooled together as birch) found on cutblocks at each study site. Residues originated from partial harvesting at Hereford Forest and clearcutting at all other sites performed in 2019 or 2020.

Leftover debris from harvesting, mostly logs from tree stems or large branches lying on the ground or in piles, were selected on cutblocks to represent a range of diameters and decay stages for each species. From each selected piece of debris, we extracted a 5 to10 cm-thick disc from the middle of the debris (i.e., avoiding the ends) using a chainsaw. We collected a total of 122 discs (73 for balsam fir; 49 for birch) and stored them at –17 °C immediately after sampling. We visually estimated the decay class of the debris in the field according to the four classes (A, B, C, D) of the wood decomposition system of Canada's National Forest Inventory (National Forest Inventory 2021) (See Table 1 in Supplementary material). Prior to laboratory analyses, bark was removed from all discs to ensure that only wood properties and chemical composition were analysed.

For each of the 122 discs, we collected two wedge-shaped subsamples, which ran from the center of the disc to its periphery. Subsamples were dried at 103 °C for 24 h. A first subsample from each disc was then used to determine wood density (kg m⁻^3^). The subsamples were weighed and then submerged in water to measure the displaced water volume, following the ASTM D2395-17 (2017) standard. The second subsample from each disc was ground into a fine powder and sieved through 425 µm and then 250 µm meshes for chemical analyses.

We quantified total carbon (C), sulfur (S) and N concentrations (%) from 1 mg of ground subsamples by dry combustion using a LECO TruMac CNS-2000 (LECO Corporation, St-Joseph, MI, USA). Samples were heated at 1350 °C for 2 min. Carbon dioxide (CO₂) was quantified by infrared detection, while nitrogen oxides (NO₂ and NOx) were measured by thermal conductivity.

To assess ash content, 2.0 g of each ground subsample was combusted at 575 ± 25 °C for 6 h. The resulting ash subsamples were then analysed for K, Ca, Mg and sodium (Na) concentrations (ppm) using inductively coupled plasma optical emission spectroscopy (Optima 7300 ICP-OES, Perkin Elmer Inc., Waltham, Mass, USA).

We determined the net calorific value of wood, expressed in energy per unit mass (MJ kg⁻^1^), on a randomly selected subset of 81 samples: 54 from balsam fir and 27 from birch. For this analysis, 0.7 g of each ground wood subsample was compressed into a tablet and combusted in an oxygen-rich atmosphere using a Parr 6400 bomb calorimeter (Parr Instrument Company, Moline, IL, USA).

We then analysed the ground wood samples using near-infrared reflectance spectroscopy (NIRS; DS2500, Foss A/S, Hillerød, Denmark) across wavelengths of 400–2500 nm. This analysis aimed to characterize the wood organic matter content. Chemometric analyses were conducted using WinISI 4.3 software (Foss A/S) to calibrate NIRS measurements and correct for instrumental errors.

A subset of all ground samples was then randomly selected and analysed using an ANKOM A200 Fiber Analyser (ANKOM Technology, Fairport, NY, USA) to quantify their neutral detergent fiber (NDF), acid detergent fiber (ADF), and acid detergent lignin (ADL) contents. NDF represents the combined content of hemicellulose, cellulose, and lignin; ADF includes cellulose and lignin; and ADL isolates the lignin fraction. Hemicellulose content was estimated by subtracting ADF from NDF, and cellulose by subtracting ADL from ADF. We then developed a calibration model using WinISI to predict NDF, ADF, and ADL concentrations across all samples based on their near-infrared (NIR) spectral characteristics. Concentrations of cellulose, hemicellulose and lignin for each sample were then deducted based on NDF, ADF and ADL concentrations. See Table 2 in Supplementary material for the mean values of all measured wood chemical composition concentrations and physical properties.

### Statistical analyses

We conducted all statistical analyses in R version 4.4.1 (R Development Core Team 2024), using a significance level of *α* = *0.05*. We first performed a factor analysis of mixed data to explore the relationships between site characteristics as expressed by bioclimatic domain and soil CEC [mol kg^−1^], wood chemical composition (including C [%], N [%], K [ppm], Ca [ppm], Mg [ppm], Na [ppm], S [%], hemicellulose [%], cellulose [%] and lignin [%]) concentrations and wood density (kg m^−3^)) of all 122 samples. This analysis allowed investigating the relationships between the quantitative variables associated with residues and the bioclimatic domain of wood source location. The analysis was conducted using the *FAMD* function 'FactoMineR' package of Lê et al. (2008), and biplots were generated to visualize and interpret the contributions of individual variables to the relationships.

We then assessed the effects of explanatory variables related to (i) the characteristics of the source location, i.e., soil CEC (at a 15–30 cm depth) and the bioclimatic domain (sugar maple–yellow birch, balsam fir–white birch, balsam fir–yellow birch), (ii) the wood species and iii) the wood decomposition stage (expressed by the decomposition class A, B, C and D) on wood properties using linear mixed-effects models. The *lmer* function from the 'lme4' package (Bates et al. 2015) was used to fit models for each wood chemical composition variable as well as for wood density and calorific value as response variables. We included the sampling site as a random factor to control for site-specific effects and account for random variation.

The general model structure was as follows:

1 $${Y}_{ij}= \alpha +{{\beta }_{1} Decomposition}_{i} +{{\beta }_{2} CEC}_{i} +({Species}_{i} \times {Domain}_{i}){+ (1|SITE}_{j})+{\varepsilon }_{ij}$$

where:

- α is the intercept, *i* index for each individual observation,
- β_1_ and β_2_ are regression coefficients for decomposition class and CEC, respectively,
- (1|SITE_j_) accounts for random effects of each site,
- ɛ_ij_ represents the residual error, assumed to be normally distributed (ɛ_ij_ ~ N (0, σ_residual_)).

When the sampling site did not explain a significant share of variance in models (e.g., variance = 0), the random effect (sampling site) was dropped from the model to avoid unnecessary complexity; this was the case for K and hemicellulose concentrations. We excluded wood S concentration from the analysis because its distribution did not meet the assumptions of the linear model.

The model for net calorific value was based on a subset of 81 observations (54 balsam fir, 27 birch); we only tested the interaction effect between species and bioclimatic domain (i.e., the decomposition class and the site soil CEC were dropped from the model).

Species-specific linear mixed models were used for lignin concentration to test the effect of decomposition, soil CEC and bioclimatic domain, due to the a priori knowledge that lignin concentration should differ significantly between balsam fir and birch, a commonly observed difference between hardwood and softwood species (Evans 1991; Toscano et al. 2017):

2 $${Y}_{ij}= \alpha +{{\beta }_{1} Decomposition}_{i} +{{\beta }_{2} CEC}_{i}+{Domain}_{i}+{\varepsilon }_{ij}$$

We used the *emmeans* function from the 'emmeans' package (Lenth et al. 2024) to calculate the estimated marginal means and performed *post-hoc* Benjamini–Hochberg contrasts tests for categorical variables (Benjamini and Hochberg 1995), i.e., wood decomposition class (A, B, C and D) and the interaction between species and bioclimatic domain. The *ggemmeans* function from the 'ggeffects' package (Lüdecke 2021) was used to produce the prediction effects for each model. Linear model assumptions were evaluated using the *check_model* function from the 'performance' package (Lüdecke et al. 2021), including homoscedasticity, normality of residuals and multicollinearity (assessed using the variance inflation factor VIF < 5).

## Results and discussion

### Relationships between wood properties, soil CEC and bioclimatic domains

**Fig. 2:**
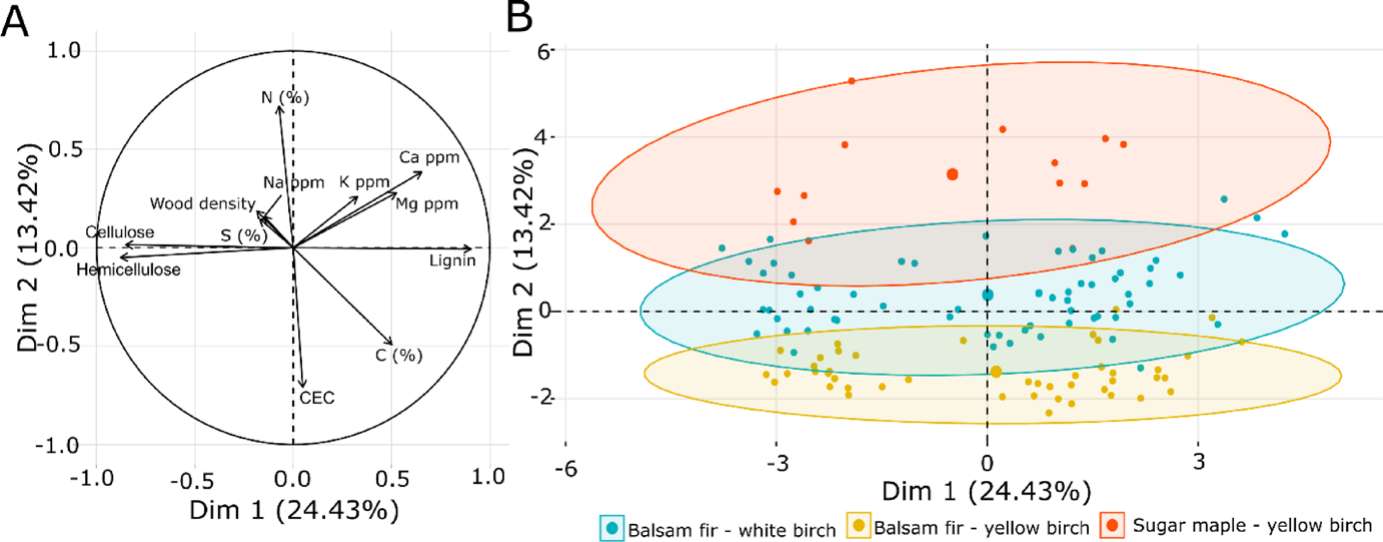
**A** Factor analysis of mixed data for quantitative variables (Carbon (C) [%], Nitrogen (N) [%], Potassium (K) [ppm], Calcium (Ca) [ppm], Magnesium (Mg) [ppm], Sodium (Na) [ppm], Sulfur (S) [%], soil CEC [mol kg^−1^], hemicellulose [%], cellulose [%], lignin [%] and wood density [kg m^−3^]) and qualitative variables (bioclimatic domain [balsam fir–white birch (*Betula papyrifera* Marsh.), balsam fir–yellow birch (*Betula alleghaniensis* Britton) and sugar maple (*Acer saccharum* Marsh.)–yellow birch]). **B** Representation of bioclimatic domain clusters and their mean observation (the ellipse containing 95% of observations and the mean in the center) within the two-dimensional space defined by the factor analysis

The factor analysis of mixed data was performed to explore relationships between wood physical and chemical characteristics and variables related to the source location of wood. The first two dimensions of the factor analysis of mixed data explained a cumulative variance among variables of 37.85% (Fig. 2A). The first dimension (24.43%) was primarily associated with differences in wood chemical composition (cellulose, hemicellulose and lignin concentrations), while the second dimension (13.42%) captured the correlations related to soil CEC, and some wood chemical elements, mainly N and C concentrations (Fig. 2A). Wood carbohydrate (cellulose and hemicellulose) concentrations were opposed to lignin concentration and aligned with the first dimension (Fig. 2A), consistent with the known trade-off between structural and energy-storage components in wood (Martin et al. 2013). Ca was positively correlated to the first two dimensions (Fig. 2A). The second dimension captured apparent differences across bioclimatic domains, particularly in relation to soil CEC, as well as wood N and C concentrations. The sugar maple–yellow birch domain appeared to be associated with higher wood N and Ca concentrations, whereas the balsam fir–yellow birch domain was strongly related to higher soil CEC and wood C concentration (Fig. 2B). The biplot of the decomposition classes did not show a clear division of each class as they were overlapping each other (See Fig. 1 in Supplementary material).

The biplot also highlighted strong correlations among wood cations (Ca, Mg, and K), suggesting co-regulation of these elements. These results indicate that while bioclimatic domains (accounting for ecological context and climatic gradient) influence soil fertility and specific wood chemical traits, they have no correlation with wood hemicellulose, cellulose, and lignin concentrations.

### Effect of bioclimatic domains and the decomposition class on wood properties

Based on the results from linear models, we observed a significant interaction between species and bioclimatic domain for most response variables, i.e., C, N, K, Ca, Mg, cellulose, and hemicellulose concentrations, as well as wood density and calorific value (Tables 3 and 4 in Supplementary material). Among these, C, cellulose and hemicellulose concentrations were the only variables consistently showing species differences within each bioclimatic domain, distinguishing balsam fir from birch wood (Fig. 2 in Supplementary material). In contrast, no significant effect of species and bioclimatic domain was observed for wood Na concentration.

The wood decomposition class was overall the second most important factor (after the species × bioclimatic domain interaction) significantly impacting wood N, K, Ca, hemicellulose and cellulose concentrations (Tables 3 and 4 in Supplementary material). Soil CEC of the wood location source had a smaller (yet significant) effect, only affecting wood cellulose concentration (Table 3 in Supplementary material). Overall, the marginal R^2^ of linear mixed models ranged from 13.0% to 60.5% (Table 3 in Supplementary material). The fit of the linear models was inconsistent across each tested value, with the highest adjusted R^2^ at 83.9% for hemicellulose and the lowest at 3.9% for birch lignin content (Table 4 in Supplementary material).

### Bioclimatic domains and species

**Fig. 3:**
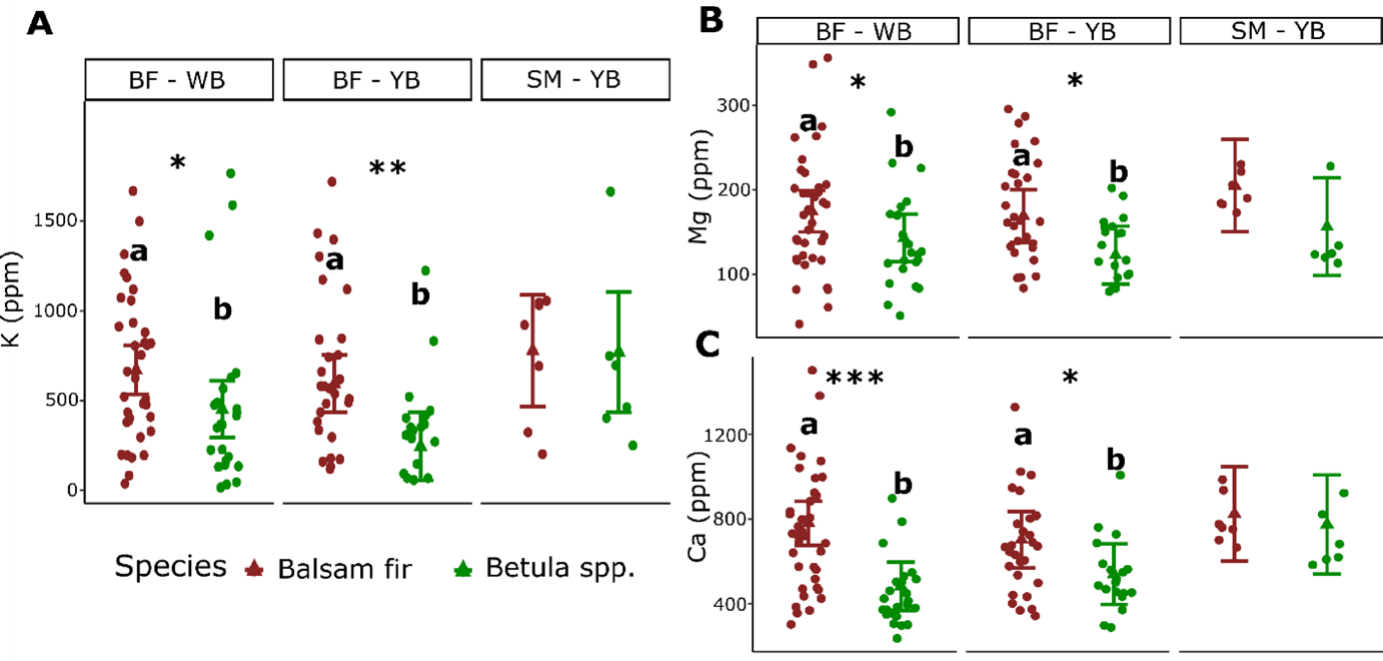
**A** Predicted potassium (K), **B** magnesium (Mg) and **C** calcium (Ca) concentrations for balsam fir (*Abies balsamea*) and birch spp. (*Betula alleghaniensis* and *Betula papyrifera*) wood samples collected from forest management residues in the balsam fir–white birch (BF–WB), balsam fir–yellow birch (BF–YB), and sugar maple–yellow birch (SM–YB) bioclimatic domains. Error bars represent standard deviations. For each combination of response variable and bioclimatic domain, statistical differences between species are indicated by asterisks (*p < 0.05, **p < 0.01, ***p < 0.001) and lowercase letters. Dots correspond to the original observations and triangles to the mean prediction

Wood concentrations of K, Mg and Ca were significantly higher in balsam fir wood than birch, within the balsam fir–white birch and balsam fir–yellow birch bioclimatic domains (Fig. 3A, 3B and 3C). Balsam fir wood K concentration was 32.5% higher in the balsam fir–white birch domain (670 ppm vs 452 ppm) and 58.7% higher in the balsam fir–yellow birch domain (593 ppm vs 245 ppm) (Fig. 3A). Balsam fir wood Mg concentration was 18.3% higher in the balsam fir–white birch domain (175 ppm for balsam vs 143 ppm for birch) and 27.2% higher (169 ppm for balsam vs 123 ppm for birch) in the balsam fir–white yellow domain. Wood Ca concentration was significantly higher for balsam fir compared to birch in the balsam fir–white birch domain (780 ppm vs 482 ppm, *p* < 0.001) and in the balsam fir–yellow birch domain (702 ppm vs 539 ppm, *p* < 0.05) (Fig. 3C). K, Mg and Ca wood concentrations did not differ between species in the sugar maple–yellow birch bioclimatic domain (Fig. 3A, 3B and 3C).

Base cations (Ca, Mg and K) showed similar patterns, with higher concentrations in balsam fir than in birch in the northern bioclimatic domains (balsam fir–white birch and balsam fir–yellow birch), but lower concentrations in the sugar maple–yellow birch domain. From a bioenergy perspective, high K and Na concentrations are undesirable, as they contribute to slagging and boiler fouling during thermochemical conversion (Elbersen et al. 2017; Miles et al. 1995). The Alkali Index proposed by Miles et al. (1995) combined alkali content (K and Na) of a fuel to its energy content to assess slagging and fouling risk. Therefore, balsam fir from northern regions may be less suitable for thermochemical conversion due to increased boiler maintenance requirements compared to birch. However, higher Ca and Mg concentrations (observed for balsam fir) can improve biomass suitability for bioenergy, as Ca increases ash melting temperatures and Mg enhances ash behavior during combustion (Elbersen et al. 2017). Overall, when considering macroelement composition alone, neither species clearly emerged as superior for thermochemical bioenergy production. Trade-offs between desirable and undesirable traits, such as K and Na versus Ca and Mg content, must be considered when matching bioenergy feedstock with conversion technology and equipment.

**Fig. 4:**
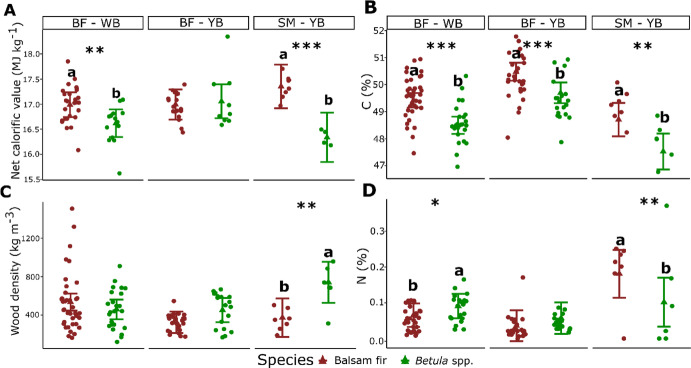
**A** Predicted net calorific value, **B** carbon (C) concentration, **C** wood density and **D** nitrogen (N) concentration, for balsam fir (*Abies balsamea*) and birch spp. (*Betula alleghaniensis* and *Betula papyrifera*) wood collected from forest management residues in the balsam fir–white birch (BF–WB), balsam fir–yellow birch (BF–YB), and sugar maple–yellow birch (ESM–YB) bioclimatic domains. Error bars represent standard deviations. For each combination of response variable and bioclimatic domains, statistical differences between species are indicated by asterisks (*p < 0.05, **p < 0.01, ***p < 0.001) and lowercase letters. Dots correspond to the original observations and triangles to the mean prediction

In two bioclimatic domains, the net calorific value of residues from balsam fir was higher than that of birch (i.e., 6.3 and 2.4% higher, respectively, in the balsam fir–white birch and the sugar maple–yellow birch domains) (Fig. 4A). The wood C concentration was significantly influenced by the interaction between species and bioclimatic domain. The magnitude of species differences remained relatively similar across bioclimatic domains, but the difference was higher in the balsam fir–white birch and balsam fir–yellow birch domains (Fig. 4B). Wood density only differed in the sugar maple–yellow birch bioclimatic domain between birch and balsam fir (740 kg m^−3^ vs 372 kg m^−3^, *p* < 0.01) and no significant difference were detected in the two other domains (Fig. 4C). We detected significant species differences in wood N concentration in the balsam fir–white birch and in the sugar maple–yellow birch domains (Fig. 4D). The mean N concentrations for balsam fir and birch were 0.07% and 0.08% in the balsam fir–white birch domain and 0.2% and 0.1% in the sugar maple–yellow birch domain, respectively (Fig. 4D).

Our measures of calorific value for birch are lower than those observed by Dupuis et al. (2020), which oscillated around 19.5 MJ kg^−1^. Singh and Kostecky (1986) reported slightly higher calorific values for regular balsam fir wood (18.75 MJ kg^−1^) than we did in the current study. In Europe, species used for bioenergy production often include European beech (*Fagus sylvatica* L.) (17.8 to 18.4 MJ kg^−1^; Piętka et al. 2019), Norway spruce (*Picea abies* (L.) Karst.) (18.62 to 20.05 MJ kg^−1^; Gendek et al. 2023) and silver fir (*Abies alba* Mill.) (18.81 to 19.47 MJ kg^−1^; Gendek et al. 2023), which have higher calorific values here than those recorded for balsam fir and birch. Black spruce (*Picea Mariana* (Mill.) BSP) and jack pine (*Pinus banksiana* Lamb.), other common Canadian tree species, have wood calorific values of 19.6 MJ kg^−1^and 19.4 MJ kg^−1^, respectively (Hosegood et al. 2011; Singh and Kostecky 1986). Thus, the calorific values of residues from balsam fir and birch appear to fall on the lower end of the range for energy release upon combustion among common commercial tree species. However, these values for fir and birch included undecayed to slightly decomposed wood sampled and the comparison with other species were undecayed wood.

Variations associated with species and bioclimatic domains from which the biomass is sourced suggest that forest biomass supply chains located in specific regions might be able to mobilise a higher energy content per unit mass of residues. Indeed, forest stand characteristics varied from one region to another, influencing both the species composition and the species-specific quantity of harvesting residues on cutblocks. Based on the coarse woody debris (CWD; diameter ≥ 3.1 cm) data from an empirical study at the same sites as ours (Canuel et al. 2024), we estimated the mean mass of harvesting residues available for balsam fir and birch within each bioclimatic domain (See Table 5 in Supplementary material). Using our species-specific calorific value, these residues correspond to a theoretical energy availability of 44.2 to 303.2 MJ ha^−1^ for balsam fir and 72.2 to 215.4 MJ ha^−1^ for birch (Table 5 in Supplementary material). The total energy potential per harvested area would likely be higher if residues from all tree species were included. Variation in estimates within and among bioclimatic domains was largely explained by differences in CWD mass per hectare, which in turn reflects wood product demand and harvesting practices (Canuel et al. 2024, 2025b, a). In practice, only a portion of this theoretical energy (e.g., 50–70%) could realistically be recovered in boreal and temperate forests because of technical constraints (e.g., accessibility, machinery efficiency) and ecological considerations (e.g., nutrient cycling, habitat retention) for residue removal (Thiffault et al. 2015).

Wood density differed between species only in the sugar maple–yellow birch bioclimatic domain, where birch had significantly higher density than balsam fir. This likely reflects the higher proportion of yellow birch in samples from this domain, which has a higher wood density compared to white birch, the more abundant birch species in northern domains. We also tested the effect of pre-harvest stem density in studied stands to see if it impacts the wood density of balsam fir and birch, but the results were not significant (result not shown). Higher material density facilitates the various stages of handling and transportation, whereas low density increases transportation and storage costs (Irmak 2019). While other studies have indicated that stand location can significantly impact wood density (Tharakan et al. 2003), it does not appear to be an important driver in our study and should therefore not unduly influence the logistics and economics of supply chains.

Patterns in wood N concentration were less consistent. From a thermochemical conversion perspective, maintaining N concentrations in woody biomass below 0.2% is important, as higher levels contribute to NOx emissions that require costly emission controls (Elbersen et al. 2015, 2017). In our study, N concentration in the two northernmost bioclimatic domains were low (~ 0.08% and ~ 0.05%) and increased to ~ 0.18% for balsam fir in the sugar maple–yellow birch domain. These values indicate that NOx emissions would likely remain relatively low when using balsam fir and birch wood (without bark) across the studied regions. Moreover, N is generally less problematic for combustion than other elements such as Cl or K, which have a more direct influence on ash behavior and boiler performance (Elbersen et al. 2017).

**Fig. 5:**
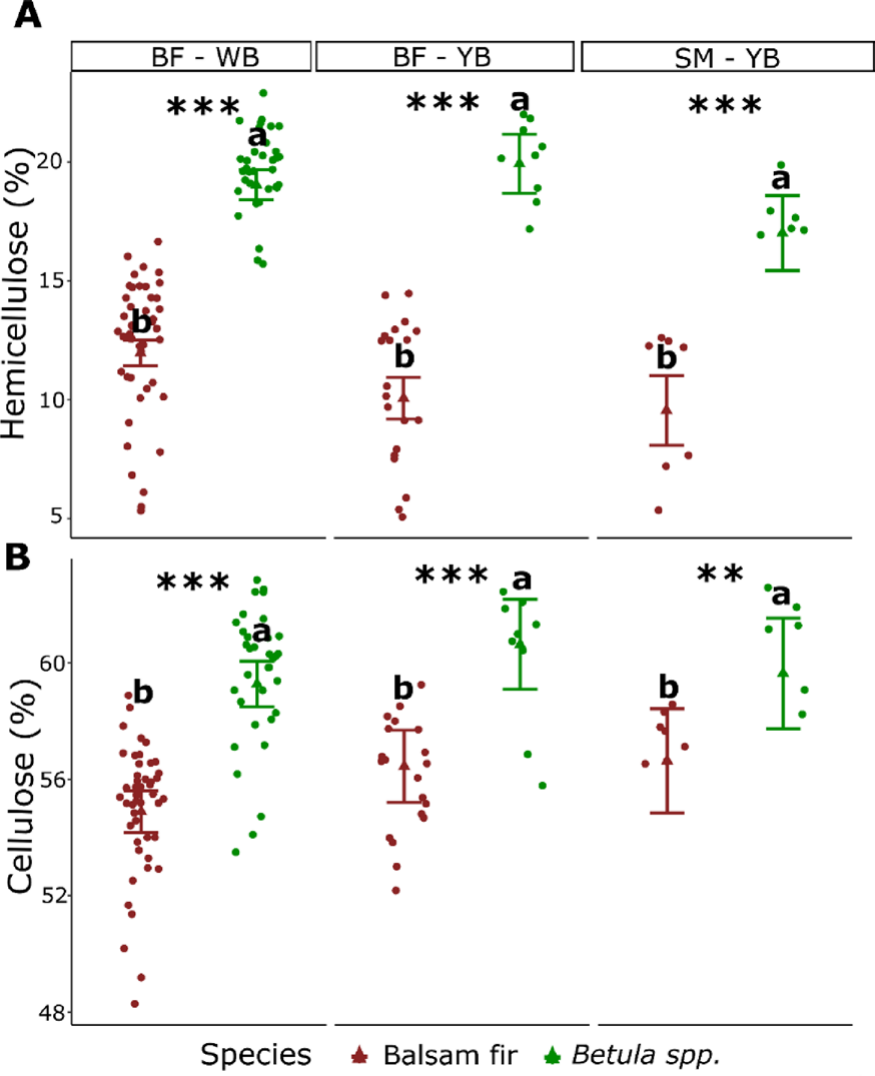
**A** Predicted hemicellulose, **B** cellulose concentrations for balsam fir (*Abies balsamea*) and birch spp. (*Betula alleghaniensis* and *Betula papyrifera*) wood samples collected from forest management residues in the balsam fir–white birch (BF–WB), balsam fir–yellow birch (BF–YB), sugar maple–yellow birch (SM–YB) bioclimatic domains. Error bars represent standard deviations. For each combination of response variable and domain, statistical differences between species are indicated by asterisks (*p < 0.05, **p < 0.01, ***p < 0.001) and lowercase letters. Dots correspond to the original observations and triangle to the mean prediction

We observed consistent significant differences in cellulose and hemicellulose wood concentrations between balsam fir and birch irrespective of the bioclimatic domain (Fig. 5). Hemicellulose concentration in balsam fir ranged from 12.6% (in balsam fir–white birch) to 8.1% (in sugar maple–yellow birch), whereas it ranged from 20.3% (in balsam fir–yellow birch) to 15.5% (in sugar maple–yellow birch) for birch (Fig. 5A).

Balsam fir consistently showed a concentration of ~ 30% lignin, while birch averaged ~ 14%. The higher lignin content in balsam fir is desirable for bioenergy production, as lignin combustion releases more energy than cellulose or hemicellulose (Elbersen et al. 2015; Lamlom and Savidge 2003). However, this higher lignin content in balsam fir could present challenges, as lignin has a broad thermochemical degradation range (160 °C–900 °C) and high lignin is less desirable for pyrolysis processes (Kandanelli et al. 2018). Lignin combustion also contributes to ash production, impacting energy conversion efficiency and reactor maintenance needs (Dupuis et al. 2020; Elbersen et al. 2017).

### Decomposition class effect on properties

**Fig. 6:**
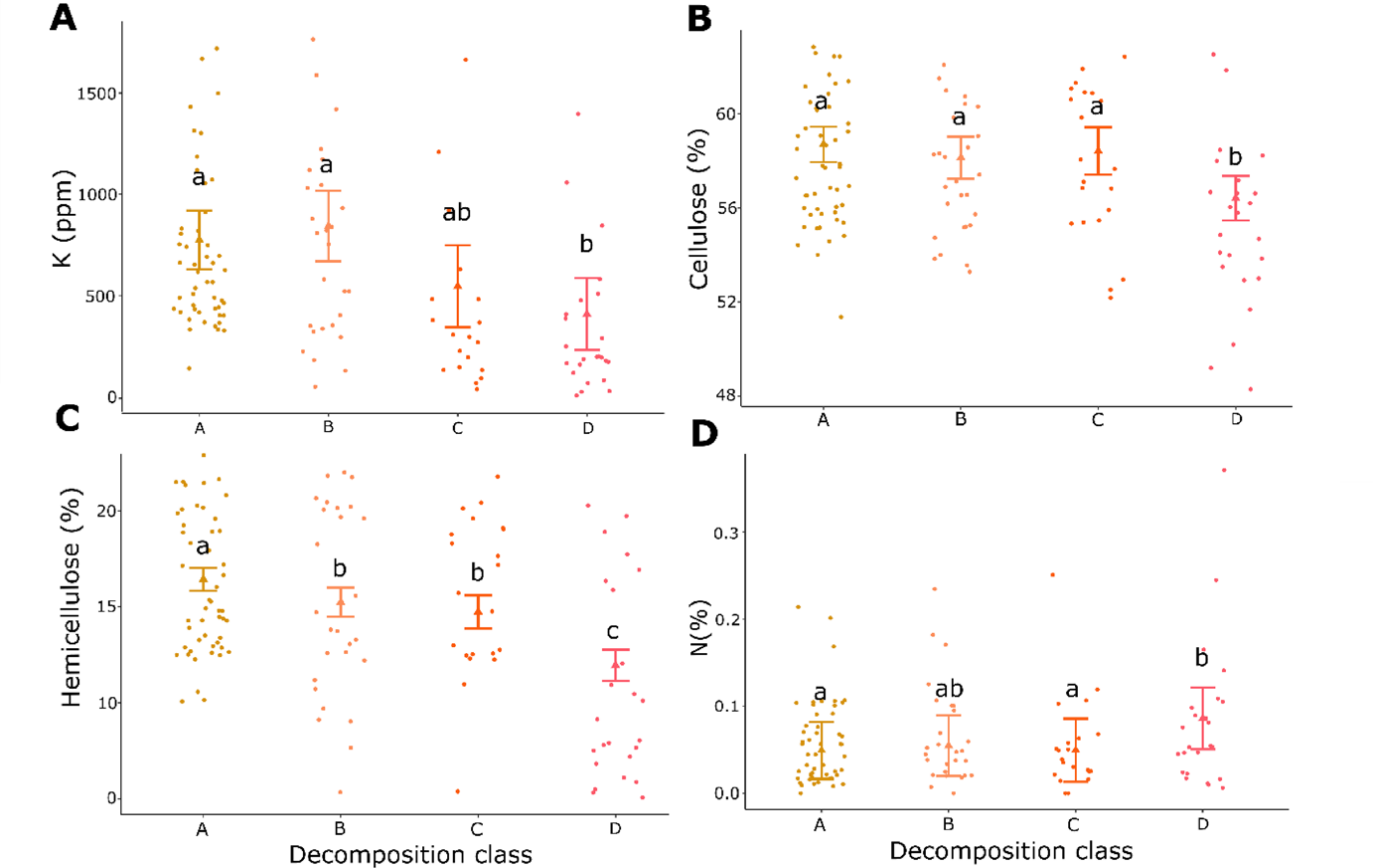
**A** Predicted potassium (K, ppm), **B** cellulose (%), **C** hemicellulose (%), **D** nitrogen (N, %) concentrations combining balsam fir (*Abies balsamea*) and birch spp. (*Betula alleghaniensis* and *Betula papyrifera*) wood samples collected from forest management residues as a function of each decomposition class (A, B, C, D). Error bars represent standard deviations. Statistical differences between decomposition classes are indicated by lowercase letters. Dots correspond to the original observations and triangles to the mean prediction

K, cellulose, and hemicellulose concentrations decreased as the wood decomposition increased from class A to D (Fig. 6A, B, C) across all sites and species. In contrast, N concentration increased with increasing decomposition stages (Fig. 6D). The decrease in K concentration went from 715 ppm in class A to 340 ppm in class D (Fig. 6A). Cellulose concentration remained consistent across classes A (58.7%), B (58.1%) and C (58.4%) and decreased in class D, i.e., the most advanced decay stage (56.4%) (Fig. 6B). For hemicellulose concentration, the highest values were observed in class A (16.4%), followed by classes B (15.2%) and C (14.7%), which were not significantly different, while class D exhibited the lowest levels (12.0%) (Fig. 6C). Finally, wood N concentration increased as apparent decomposition advanced as classes A and C had similar levels and were different from D, while class B was intermediate and not significantly different from other classes (Fig. 6D). Moreover, the decomposition class steadily increased the Ca concentration, from a mean value of 588 ppm (class A) to 772 ppm (class D) (Fig. 3 in Supplementary material).

In the context of bioenergy production, wood decomposition generally leads to undesirable changes, such as reduced hemicellulose and cellulose content in wood and increased N concentration—both of which are detrimental to thermochemical conversion processes (Elbersen et al. 2015). Previous studies have highlighted the negative influence of decomposition on Ca, K, N, hemicellulose, and cellulose for bioenergy (Kumar and Wyman 2009; Shi et al. 2008). Wood decomposition is primarily driven by fungi, which alter its chemical composition and consequently, its calorific value (Gendek et al. 2023; Piętka et al. 2019). Fungal wood decay is generally classified into two main types: white rot and brown rot, both of which degrade cellulose, hemicellulose, and lignin through enzymatic activity (Piętka et al. 2019; Vidholdová et al. 2022). White rot, caused by basidiomycetes, decomposes cellulose, hemicellulose, and lignin either simultaneously or selectively. *Rhodofomes roseus* (Alb. & Schwein.) Kotl. & Pouzar causes brown rot which primarily degrades cellulose and hemicellulose while leaving lignin largely intact (Gendek et al. 2023). Moreover, the decomposition process can increase the amount of ash produced during thermochemical conversion, which is undesirable (Dupuis et al. 2020).

Our study found a decrease in K concentration as decomposition progressed, dropping by 375 ppm between decomposition class A (715 ppm) and class D (340 ppm). This reduction in K could help mitigate sintering and fouling issues during thermochemical conversion (Elbersen et al. 2017). Therefore, degraded wood from birch and balsam fir, which might not find markets in conventional wood industries, remain suitable for bioenergy use. However, the measured K concentrations displayed a wide variability across our samples, ranging from < 15 ppm to > 4000 ppm, a pattern also observed by Paré et al. (2013). Consequently, relying solely on the reduction of K concentration as a criterion for residue collection would be too simple, as optimal feedstock should account for multiple wood property changes that occur during decomposition (Dupuis et al. 2020; Gendek et al. 2023; Piętka et al. 2019). An extended biomass procurement timeframe following harvesting, combined with suitable feedstock selection, offers the advantage of reducing wood moisture in harvest residues (Väätäinen et al. 2021), but may also provide additional ecological benefits (Manolis et al. 2019). Indeed, as residues decompose, they gradually lose their bark (in addition to shedding foliage), which contains higher micronutrient concentrations than wood (Manolis et al. 2019) and may thus contribute to maintain soil fertility.

As expected, we observed reductions in cellulose and hemicellulose concentrations, primarily in the most advances decomposition class (D), consistent with brown rot fungal degradation of these components. Specifically, carbohydrate concentration (cellulose + hemicellulose) decreased by 9%, from 74.9% in class A to 68.2% in class D. Carbohydrate concentration above 65% is considered desirable for thermochemical biomass production (Elbersen et al. 2015). The samples we analysed thus remained within an acceptable range.

Wood decomposition slightly increased N concentration in both birch and balsam fir wood, though values remained low (0.08%–0.11%). These levels fall within acceptable thresholds for thermochemical conversion, ensuring minimal NOx emissions. Our results differ from those of Dupuis et al. (2020), who reported no effect of decay level on white birch wood properties; however, their small sample size (N < 10) may have limited statistical power.

Combining white and yellow birch in our analyses reduced species-specific precision, but it allowed for a broader assessment of regional variation in birch wood properties. Since our study mainly focused on wood from leftover logs and large branches (without distinction between stem and branch wood samples), future research should investigate the specific physical properties and chemical composition of stems vs. branches and those of other tree components—such as bark, foliage, and fine branches—to provide a more comprehensive evaluation of balsam fir and birch bioenergy potential (Elbersen et al. 2017).

## Conclusion

Studying the chemical and physical properties of balsam fir and birch wood from forest management residues revealed that both the source location and the state of decomposition of wood significantly influenced wood properties relevant to thermochemical conversion. Notably, balsam fir exhibited slightly higher net calorific values than birch in two of the studied bioclimatic domains. In contrast, balsam fir consistently showed higher K concentrations, which may be problematic for thermochemical conversion, as elevated K levels can be associated with sintering, slagging, and boiler fouling. Our findings confirm that field-assessed decomposition provides a rapid and effective method for evaluating the bioenergy potential of residues, particularly for highly decayed wood. From a thermochemical conversion standpoint, wood decomposition was beneficial in reducing K levels while maintaining cellulose, hemicellulose, and N concentrations within desirable thresholds for bioenergy applications. Moreover, allowing some degree of decomposition on-site may help maintain soil fertility, as partially decomposed logs could provide carbon and nutrients to soils. Considering the combined effects of wood decomposition and bioclimatic conditions across all measured wood properties, both balsam fir and birch species appear to be suitable feedstocks for thermochemical biomass conversion. Other feedstock analyses such as elemental composition in hydrogen (H) and oxygen (O) will further help optimize the choice of conversion pathways. Integrating the collection of forest management residues for bioenergy can enhance value creation while contributing to the stability of the overall wood supply chain.

## Supplementary Information

Below is the link to the electronic supplementary material.

Supplementary Material 1

## Data Availability

Data are available on the FigShare platform: 10.6084/m9.figshare.30086005.v1.
