# Supplementary material for: Wood chemical composition of forest management residues for bioenergy
Source: Wood Sci Technol. 2026 Mar 7;60(3):39. doi: 10.1007/s00226-026-01760-z (PMC12966221; doi:10.1007/s00226-026-01760-z)
Supplement: Supplementary file 1 — Supplementary Material 1 [file 226_2026_1760_MOESM1_ESM.docx]

# Supplementary material

Table 1. Adapted decomposition class from Canada's National Forest Inventory for wood.

| Wood characteristics | **A Class**  **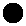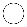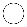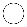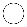** | **B Class 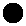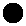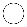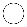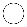** | **C Class 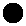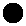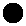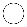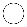** | **D Class 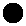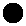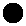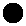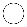** |
| --- | --- | --- | --- | --- |
| Wood texture | Hard and intact | Intact, hard et partially decomposed | Hard, partially decomposed | Decomposed with center still hard |
| Branch | Present | Some missing | Absent | Absent |
| Bark | Intact | Intact or partially present | Trace | Absent |
| Shape | Round | Round | Round | Ovoid |

Table 2. Wood chemical composition and physical properties were evaluated on 122 wood sample with the standard deviation (±), in bioclimatic domains (balsam fir–white birch, balsam fir–yellow birch, and sugar maple–yellow birch) for balsam fir (Abies balsamea) and birch (Betula alleghaniensis and Betula papyrifera), the number of sample (N), wood density (kg m^-3^), carbon (C, %), nitrogen (N, %), calcium (Ca, ppm), potassium (K, ppm), magnesium (Mg, ppm), sodium (Na, %), sulfur (S, %), cellulose (cell, %), hemicellulose (Hem, %), lignin (Lig, %) and net calorific value (MJ kg^-1^). *Analysed only on 81 samples.

| Bioclimatic domains | Species | N | Wood density | C | N | Ca | K | Mg | Na | S | Cell | Hem | Lig | Net Calorific value* |
| --- | --- | --- | --- | --- | --- | --- | --- | --- | --- | --- | --- | --- | --- | --- |
| Balsam fir – white birch | Balsam fir | 47 | 531±305 | 49.5±0.8 | 0.06±0.03 | 746±281 | 884±888 | 173±71 | 14±13 | 0.005±0.009 | 54±2 | 12.4±3.0 | 30.5±1.5 | 17.0±0.4 |
|  | Birch | 24 | 455±204 | 48.6±0.7 | 0.08±0.04 | 450±157 | 469±473 | 142±56 | 19±13 | 0.012±0.027 | 60±3 | 19.5±1.9 | 13.2±1.5 | 16.6±0.4 |
| Balsam fir – yellow birch | Balsam fir | 28 | 326±87 | 50.3±0.8 | 0.03±0.03 | 681±230 | 662±430 | 177±61 | 29±14 | 0.004±0.005 | 56±2 | 10.8±2.8 | 29.6±1.5 | 17.0±0.3 |
|  | Birch | 19 | 465±177 | 49.4±0.8 | 0.05±0.02 | 534±170 | 361±279 | 131±37 | 32±15 | 0.004±0.005 | 60±2 | 20.0±1.2 | 13.2±1.3 | 17.1±0.6 |
| Sugar maple – yellow birch | Balsam fir | 7 | 348±112 | 49.2±0.6 | 0.19±0.09 | 796±119 | 752±359 | 198±21 | 38±17 | 0.006±0.005 | 58±1 | 10.0±3.1 | 30.1±2.0 | 17.4±0.2 |
|  | Birch | 6 | 709±224 | 47.9±0.7 | 0.11±0.14 | 706±136 | 704±505 | 141±43 | 52±14 | 0.004±0.004 | 61±2 | 17.8±1.1 | 14.5±0.4 | 16.3±0.2 |

Table 3. Linear mixed model formulation with the response variables (wood density (kg m^-3^), carbon (C, %), nitrogen (N, %), calcium (Ca, ppm), magnesium (Mg, ppm), sodium (Na, ppm), cellulose (%), Net calorific value (MJ kg^-1^). The explanatory variables are decomposition classes (A, B, C, D), Cation exchange complex (CEC) and the interaction between species (balsam fir (Abies balsamea) and birch (Betula alleghaniensis and Betula papyrifera)) and bioclimatic domains (balsam fir – white birch, balsam fir – yellow birch, sugar maple – yellow birch), and the site as the random effect. The number of parameter (K) the model fit estimates with the marginal R² and conditional R² are shown. Bold character and asterisks indicate significant effect (p < 0.05*, p < 0.01**, p < 0.001***).

| Linear mixed model formulation | K | Random effect | Marginal R² | Conditional R² |
| --- | --- | --- | --- | --- |
| Wood Density ~ Decomposition class + CEC + **Species : Domain**** | 12 | 1647.1 | 0.153 | 0.181 |
| C ~ Decomposition class + CEC + **Species : Domain***** | 12 | 0.01 | 0.497 | 0.506 |
| N ~ **Decomposition class*** + CEC + **Species : Domain**** | 12 | <0.01 | 0.407 | 0.557 |
| Ca ~ **Decomposition class*** + CEC + **Species : Domain***** | 12 | 4102.3 | 0.295 | 0.356 |
| Mg ~ Decomposition class + CEC + **Species : Domain*** | 12 | 151.9 | 0.130 | 0.168 |
| Na ~ Decomposition class + CEC + Species : Domain | 12 | 87.1 | 0.295 | 0.537 |
| Cellulose ~ **Decomposition class*** + **CEC*** + **Species : Domain***** | 12 | 0.13 | 0.605 | 0.618 |
| Net calorific value ~ **Species : Domain***** | 8 | 0.04 | 0.281 | 0.458 |

Table 4. Linear model formulation with the response variables (potassium (K, ppm), hemicellulose (%), birch lignin (%) and balsam fir lignin (%). The explanatory variables are decomposition classes (A, B, C, D), Cation exchange complex (CEC) and the interaction between species (balsam fir (Abies balsamea) and birch (Betula alleghaniensis and Betula papyrifera)) and bioclimatic domains (balsam fir – white birch, balsam fir – yellow birch, sugar maple – yellow birch). The number of parameter (K), the model fit estimates with the adjusted R² and R² of each model are shown. Bold character and asterisks indicate significant effect (p < 0.05*, p < 0.01**, p < 0.001***).

| Linear model formulation | K | R² adjusted | R² |
| --- | --- | --- | --- |
| K ~ **Decomposition class**** + CEC + **Species : Domain**** | 10 | 0.206 | 0.267 |
| Hemicellulose ~ **Decomposition class** + CEC + **Species : Domain***** | 10 | 0.839 | 0.851 |
| Birch Lignin ~ Decomposition class + CEC + **Domain*** | 8 | 0.039 | 0.159 |
| Balsam fir Lignin ~ Decomposition class + CEC + Domain | 8 | 0.123 | 0.196 |


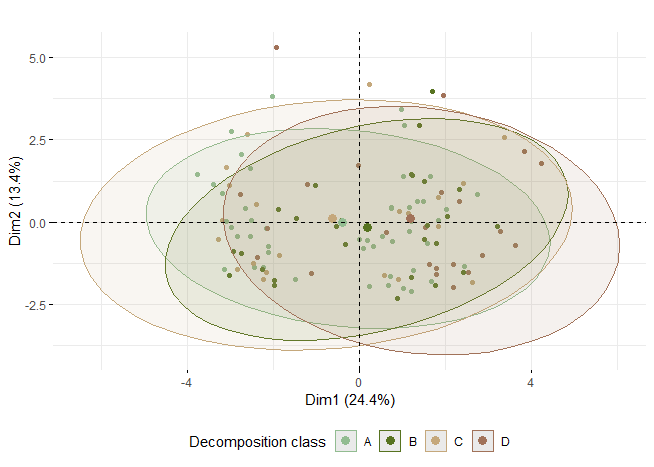


Figure 1. Factor analysis of mixed data, representing wood properties (Carbon (C) [%], Nitrogen (N) [%], Potassium (K) [ppm], Calcium (Ca) [ppm], Magnesium (Mg) [ppm], Sodium (Na) [ppm], Sulfur (S) [%], hemicellulose [%], cellulose [%], lignin [%] and wood density [kg m^-3^]) and soil CEC [mol kg^-1^]. The factor analysis is clustered by decomposition class (A, B, C, D) and their mean observation (the bigger dot in the ellipse center) within the two-dimensional space defined by the factor analysis.


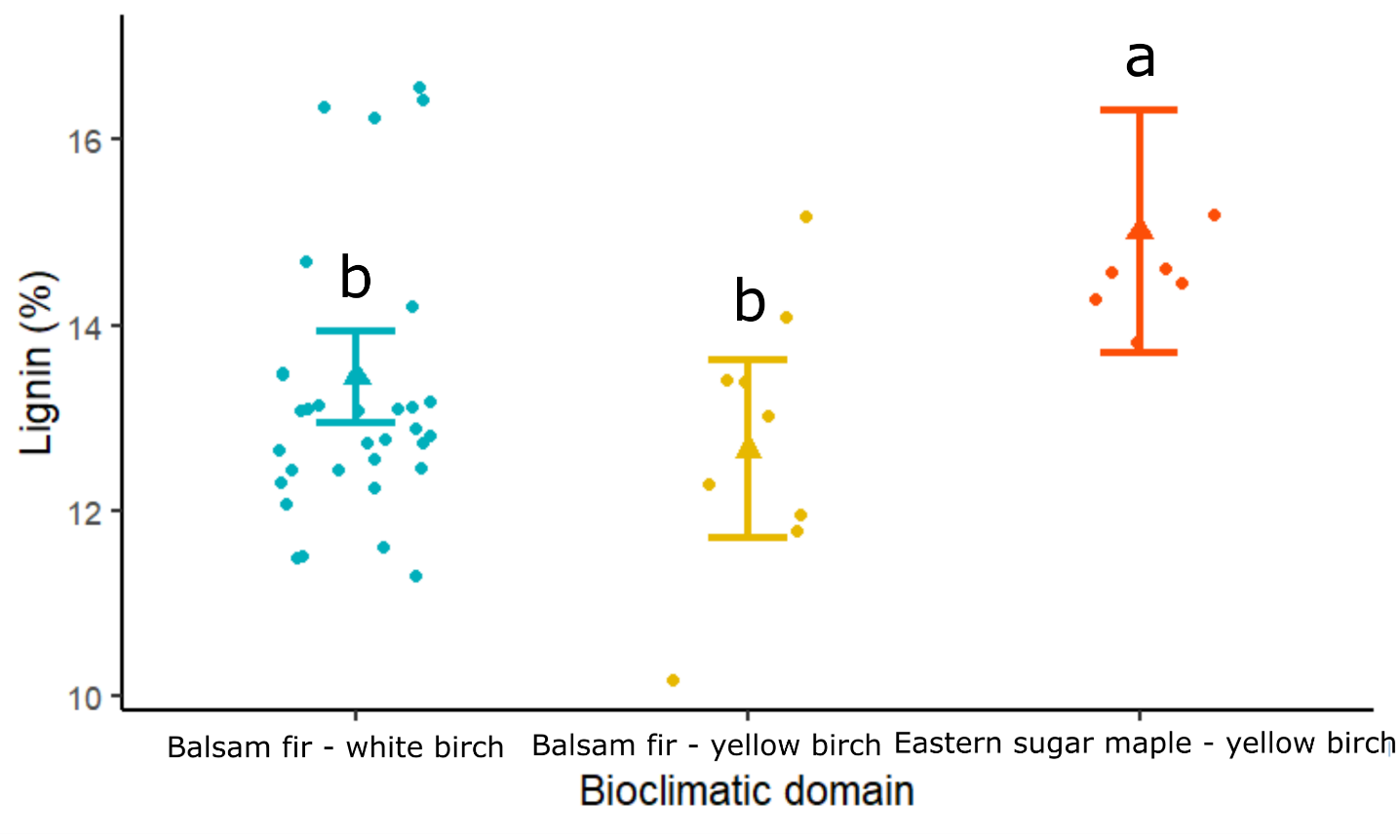


Figure 2. Predicted lignin concentrations for birch (Betula alleghaniensis and Betula papyrifera) wood samples collected from forest management residues in the balsam fir – white birch, balsam fir – yellow birch, sugar maple – yellow birch bioclimatic domains. Error bars represent standard deviations. For each combination of response variables and domain, statistical differences between species are indicated by lowercase letters. Dots correspond to the original observations and triangle to the mean prediction.


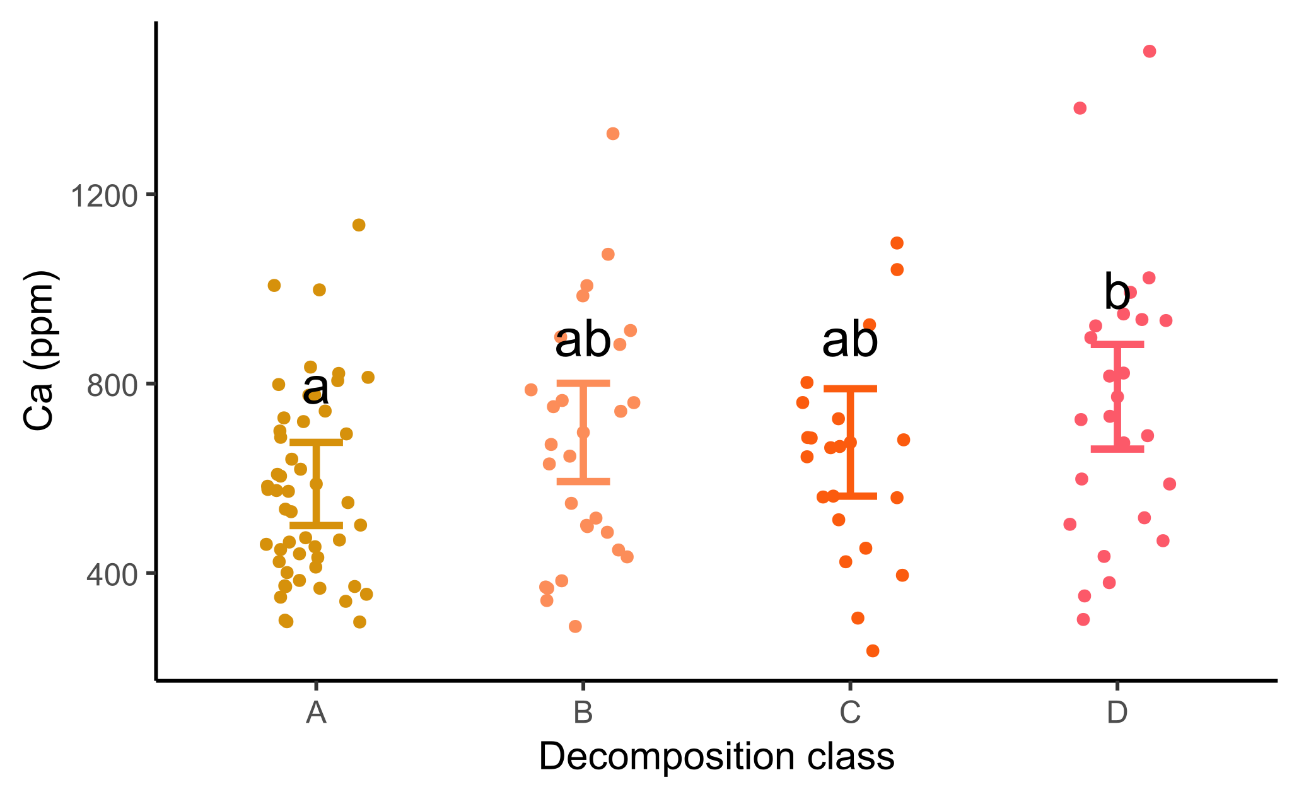


Figure 3. Predicted calcium concentration for balsam fir (Abies balsamea) and birch (Betula alleghaniensis and Betula papyrifera) wood samples collected from forest management residues as a function of each decomposition class (A, B, C, D). Error bars represent standard deviations. Statistical differences between decomposition classes are indicated by lowercase letters and dots correspond to the original observations.

Table 5: Mean energy based on the coarse woody debris mass (CWD, diameter: ≥3.1 cm) available per hectare and the net calorific power. Bioclimatic domain (balsam fir – white birch, balsam fir – yellow birch, sugar maple – yellow birch), species (balsam fir (Abies balsamea) and birch (Betula alleghaniensis and Betula papyrifera)), N mass (the number of sampling plots used to estimate coarse woody debris mass), coarse woody debris mass (CWD), N net calorific power (number of replicates used to determine net calorific power), Net calorific power, Mean energy. The CWD was sampled following Canada’s National Forest Inventory (2008) guidelines and data are from Canuel et al. (2024) using only harvesting scenarios where no biomass collection was performed during operations. The CWD mass per hectare was estimated using Canada’s National Forest Inventory (2021) compilation procedures. Standard deviations were obtained by error propagation, taking into account the number of plots and replicates from both mass and net calorific power datasets.

| Bioclimatic domain | Species | N mass | CWD (kg ha^-1^ ± SD) | N net calorific power | | Net calorific power (MJ kg^-1^ ± SD) | Mean energy (MJ ha^-1^ ± SD) |
| --- | --- | --- | --- | --- | --- | --- | --- |
| Balsam fir – white birch | Birch | 31 | 8.39 ± 6.52 | | 14 | 16.6 ± 0.4 | 139.1 ± 108.3 |
|  | Balsam fir | 31 | 17.84 ± 10.51 | | 27 | 17.0 ± 0.4 | 303.2 ± 178.8 |
| Balsam fir – yellow birch | Birch | 16 | 12.62 ± 10.44 | | 9 | 17.1 ± 0.6 | 215.4 ± 178.5 |
|  | Balsam fir | 16 | 9.63 ± 9.27 | | 19 | 17.0 ± 0.3 | 163.4 ± 157.4 |
| Eastern sugar maple – yellow birch | Birch | 16 | 4.42 ± 5.26 | | 4 | 16.3 ± 0.2 | 72.2 ± 86.0 |
|  | Balsam fir | 16 | 2.54 ± 4.03 | | 8 | 17.4 ± 0.2 | 44.2 ± 69.9 |
